# Histopathological Defects in Intestine in Severe Spinal Muscular Atrophy Mice Are Improved by Systemic Antisense Oligonucleotide Treatment

**DOI:** 10.1371/journal.pone.0155032

**Published:** 2016-05-10

**Authors:** Palittiya Sintusek, Francesco Catapano, Napat Angkathunkayul, Elena Marrosu, Simon H. Parson, Jennifer E. Morgan, Francesco Muntoni, Haiyan Zhou

**Affiliations:** 1 Dubowitz Neuromuscular Centre, Institute of Child Health, University College London, London, United Kingdom; 2 Division of Gastroenterology and Hepatology, Department of Pediatrics, Faculty of Medicine, King Chulalongkorn Memorial Hospital, Chulalongkorn University, Bangkok, Thailand; 3 Department of Pathology, Ramathibodi Hospital, Mahidol University, Bangkok, Thailand; 4 Institute of Medical Sciences, University of Aberdeen, Foresterhill, Aberdeen, United Kingdom; 5 Euan MacDonald Center for Motor Neuron Disease Research, University of Edinburgh, Edinburgh, United Kingdom; Iowa State University, UNITED STATES

## Abstract

Gastrointestinal (GI) defects, including gastroesophageal reflux, constipation and delayed gastric emptying, are common in patients with spinal muscular atrophy (SMA). Similar GI dysmotility has been identified in mouse models with survival of motor neuron (SMN) protein deficiency. We previously described vascular defects in skeletal muscle and spinal cord of SMA mice and we hypothesized that similar defects could be involved in the GI pathology observed in these mice. We therefore investigated the gross anatomical structure, enteric vasculature and neurons in the small intestine in a severe mouse model of SMA. We also assessed the therapeutic response of GI histopathology to systemic administration of morpholino antisense oligonucleotide (AON) designed to increase SMN protein expression. Significant anatomical and histopathological abnormalities, with striking reduction of vascular density, overabundance of enteric neurons and increased macrophage infiltration, were detected in the small intestine in SMA mice. After systemic AON treatment in neonatal mice, all the abnormalities observed were significantly restored to near-normal levels. We conclude that the observed GI histopathological phenotypes and functional defects observed in these SMA mice are strongly linked to SMN deficiency which can be rescued by systemic administration of AON. This study on the histopathological changes in the gastrointestinal system in severe SMA mice provides further indication of the complex role that SMN plays in multiple tissues and suggests that at least in SMA mice restoration of SMN production in peripheral tissues is essential for optimal outcome.

## Introduction

Spinal muscular atrophy (SMA) is one of the most common genetic diseases in childhood and the leading genetic cause of infant mortality [1]. It is characterized by progressive degeneration of spinal motor neurons leading to proximal skeletal muscle atrophy and paralysis. SMA is caused by functional loss of the survival of motor neuron (SMN) protein resulting from homozygous genomic deletion or mutations of the *survival of motor neuron 1* (*SMN1)* gene. There are two *SMN* genes in humans, the telomeric *SMN1* and its centromeric homolog *SMN2*, a result of intrachromosomal duplication of 5q13. *SMN1* differs from *SMN2* by several exonic and intronic single nucleotide polymorphisms, without any amino acid substitution. However, a single nucleotide (C to T) variation at position 6 in exon 7 affects the efficiency of splicing of this exon in the *SMN2* gene, leading to approximately 90% of transcripts lacking exon 7 [2,3]. The resulting truncated protein is nonfunctional and unstable. The remaining 10% fully transcribed product is not sufficient to compensate for the loss of the *SMN1* gene. The copy numbers of *SMN2* gene, which vary in the general population, are important in modulation of disease severity [4–6].

SMA is currently incurable, however significant progress in the development of experimental therapies has been achieved in recent years. Promising results from preclinical studies in animal models on antisense oligonucleotide (AON) therapy [7–10], small molecular therapy [11,12] and adenoviral vector mediated *SMN1* gene therapy [13–16] have facilitated the application of these therapeutic approaches in clinical trials. Intrathecal administration of Nusinersen (IONIS-SMNRx), an 18-mer AON in 2’-O-2-methoxyethyl (MOE) phosphorothioate chemistry that targets the intronic splicing silencer N1 element (ISS-N1) in intron 7 of *SMN2* gene, in severe SMA infant is now in phase III clinical trials with encouraging data in safety and clinical outcome measurement from the previous phase I clinical trial [17]. Small molecules and *SMN1* gene therapy are both in the early phases of clinical trials (www.clinicaltrials.org. ID: NCT02268552; NCT02240355 and NCT02122952).

SMN protein is ubiquitously expressed. While SMA has traditionally been classified as a selective lower motor neuron disease with spinal motor neurons in the anterior horn being the primary pathological target, an increasing number of clinical and experimental reports suggest that pathologies in peripheral systems could contribute to the disease progression, especially in cases at the severe end of the clinical spectrum [18, 19]. It is therefore important to characterize the involvement of different peripheral organs in SMA.

Children with SMA can suffer from a variety of functional gastrointestinal (GI) complications, such as gastroesophageal reflux, constipation, abdominal distension and retarded gastric emptying [20]. Similar GI functional defects have been reported in mice with Smn deficiency, including constipation, delayed gastric emptying, slow intestinal transit and reduced colonic motility [21]. Vascular defects have also been reported in severe cases of SMA [22,23] and in a variety of transgenic mouse models of SMN deficiency [24–26]. Digital necrosis and distal vascular thrombosis have also been reported in severe SMA infants [22,23]. Our group and others recently reported decreased vascular density in skeletal muscle biopsies and spinal cord tissues in the severe SMA transgenic mice [26,27]. We have also demonstrated defective microvascular development in skeletal muscle biopsies in young SMA patients [27]. We therefore hypothesized that the previously reported GI functional abnormalities in SMA mice could be related to a vascular abnormality in the SMA mice.

In this study, we show significant alterations in gross and microscopic anatomy of the GI tract in SMA mice, including a decrease in vascular density, and an increase in the number of enteric neurons and macrophages in small intestine. In addition, we show rescue of these histopathological defects in SMA mice in response to systemic AON treatment via administration of the therapeutic morpholino antisense oligomer PMO25. Our findings provide compelling evidence of the involvement of gastrointestinal tract in SMA, at least in this mouse model, which might well have implications for future therapeutic development.

## Materials and Methods

### Animals

The Taiwanese SMA transgenic mice FVB.Cg-Tg(SMN2)2Hung *SMN1*^*tm1/Hung*^ /J, originally created by Hsieh-Li et al. [28], were purchased from Jackson Laboratory (TJL005058; Jackson Laboratory, Bar Harbor, ME). Mice were bred and experimental procedures were performed according to protocols approved by University College London (London, UK) Biological Services and UK Home Office under the Animals (Scientific Procedures) Act 1986. The severe SMA mice, with genotype of (*SMN2*)_2_^+/-^; *Smn*^-/-^, have two copies of human *SMN2* transgene and homozygous knockout of endogenous mouse *Smn*, are referred to **SMA** mice in this study. The heterozygous non-phenotypic control mice, with genotype of (*SMN2*)_2_^+/-^; *Smn*^+/-^, are referred to **control** mice in this study.

### AON treatment in SMA mice

The AON used in this study is a 25-mer morpholino antisense oligomer, PMO25, which is designed to augment the splicing of exon7 in *SMN2* gene by targeting the ISS-N1 element in intron 7. This therapeutic target was originally identified by Singh *et al* [29], and has been approved to be the most efficient AON target so far in augmenting exon 7 splicing in *SMN2* gene [7–10]. We have previously reported the successful rescue of the severe SMA mouse model after PMO25 treatment [10,30]. PMO25 was synthesized by Gene Tools (Philomath, OR) for research use only. SMA mice were injected with a single dose of 40 μg/g morpholino antisense oligomer PMO25 subcutaneously at postnatal day 0 (PND 0). The PMO25 treated severe SMA mice are referred to **SMA+PMO25** in this study. Subcutaneous administrations were injected into the upper part of the back using a 10μl glass capillary (Drummond Scientific Company, Pennsylvania).

### Intestine tissue processing

Mice were weighed before being culled by a schedule 1 procedure at PND10 where PND0 is designated as the day of birth. The entire intestine was collected and its length was measured. The whole bowel length was defined as the length from proximal duodenum to anal region. Small bowel was measured from proximal duodenum to ileocecal junction. The proximal duodenum (1 cm from pyloric region) and distal ileum (1 cm from ileocecal junction) were dissected and embedded in optimal cutting temperature (OCT, CellPath, UK) in a mould and frozen in liquid nitrogen-cooled iso-pentane. Adjacent duodenum segments were snap frozen in dry ice and stored at -80°C for RNA extraction and western blotting procedures.

### Histopathology

At least five 3 μm transverse and longitudinal cryosections from the duodenum and ileum of each mouse, taken at 60 μm intervals, were prepared using a cryostat (Leica, Germany) and were stained with haematoxylin and eosin (H&E). Slides were viewed under a light microscope (Axioplan 2, Germany) (5× objective, 0.15NA and 20× objective, 0.5NA). Images were captured using AxioCamHRc (Rev3) and a minimum of 10 fields per section were evaluated.

### Immunohistochemistry

Sections were cut at 10 μm and randomly oriented on the slide. Five cryosections at 50 μm intervals from each mouse intestine were used for staining. Cryosections were dried for 20 minutes then fixed in 4% paraformaldehyde (PFA) for 20 minutes and rinsed in phosphate-buffered saline (PBS) for 2 minutes at room temperature. All sections were subjected to immunohistochemical analysis using primary and secondary antibodies listed in Table 1.

**Table 1:** Antibodies used in immunohistochemical analysis.

Antibodies	Host	Dilution	Catalogue number and source
**Primary antibodies**			
von Willebrand factor (vWF)	Rabbit	1:500	AB7356, Merck Millipore
Protein Gene Product 9.5 (PGP9.5)	Rabbit	1:100	Rb-9202, Neomarkers
Smooth Muscle Actin	Mouse	1:100	M0851, Dako
F4-80	Rat	1:50	MCA497GA, AbD Serotec
**Secondary antibodies**			
Alexa Fluoro®594 Anti-rabbit IgG (H+L)	Goat	1:500	A11037, Invitrogen
Alexa Fluoro®488 Anti-mouse IgG (δ2a)	Goat	1:500	A21131, Invitrogen
Alexa Fluoro®594 Anti-rat IgG (H+L)	Goat	1:500	A11007, Invitrogen

For PGP9.5 primary antibody staining, sections were subjected to antigen retrieval by boiling slides in 20mM citrate buffer (Thermo Scientific) for 15 minutes, and blocked in PBS/M.O.M (mouse IgG blocking reagent, Vector) for 1 hour at room temperature. After incubation with primary antibodies at room temperature for 1 hour, slides were rinsed in PBS/0.03% Triton for 2 minutes twice followed by incubation in secondary antibody for 1 hour. Slides were then rinsed twice with PBS/0.03% Triton for 2 minutes and mounted with hydromount (National Diagnostics) and DAPI (1:1500, Hoechst 33342, ThermoFisher Scientific) for nuclear staining.

### Imaging and quantification

Images were captured on a fluorescence microscope (Leica DM4000B, Germany) (HCX PL fluotar 2x/0.50 PH2) using Metamorph software (Molecular Device, Palo Alto, CA). A minimum of 10 equidistant images per transverse section were collected from the entire gut tube wall, which were used for all subsequent analysis. All images were analyzed and quantified using imageJ software (http://imagej.nih.gov/ij/). For vascular density, a region of interest of intestinal tissue was identified by DAPI staining and the number of vWF positive pixels was quantified and expressed as blood vessel density (pixels per unit area). In a similar manner the calculation of PGP9.5 positive pixels per unit area was used to indicate the density of ganglia of the enteric nervous system seen as myenteric or Auerbach’s plexuses, which lie along the neuromuscular ridge of the intestinal wall. The total numbers of PGP9.5 positive neurons along the gut tube walls in the transverse sections were counted and a mean was calculated. The total numbers of F4-80 positive macrophages present in each section were counted and mean was calculated per mouse.

### RT-PCR and western blotting

Duodenum samples were homogenized using a Precellys Homogenizer (Berlin Technologies) in RLT lysis buffer using RNeasy Mini Kit (Qiagen, Chatsworth, CA). Total RNA extraction and reverse-transcription were performed as described previously [10,31]. The ratio of full-length *SMN2* to *Δ7 SMN2* transcripts was quantified by quantitative real-time RT-PCR. The expression of human SMN protein was measured by western blotting as previously described [31]. β-tubulin was used as loading control in all samples. Blots were developed with an enhanced chemiluminescence detection kit (Bio-Rad, California, USA). Semi-quantification of band intensity was analyzed by imageJ software.

### Statistical analysis

Data are presented as mean ± standard error of the mean (mean ± SEM). One-way ANOVA and post *t*-test were used to determine statistical significance when comparing 2 and 3 groups of mice. GraphPad Prism 5.0 software was used for statistical analysis and graph design.

## Results

### Decreased body weight and bowel length in SMA mice

**Fig 1:**
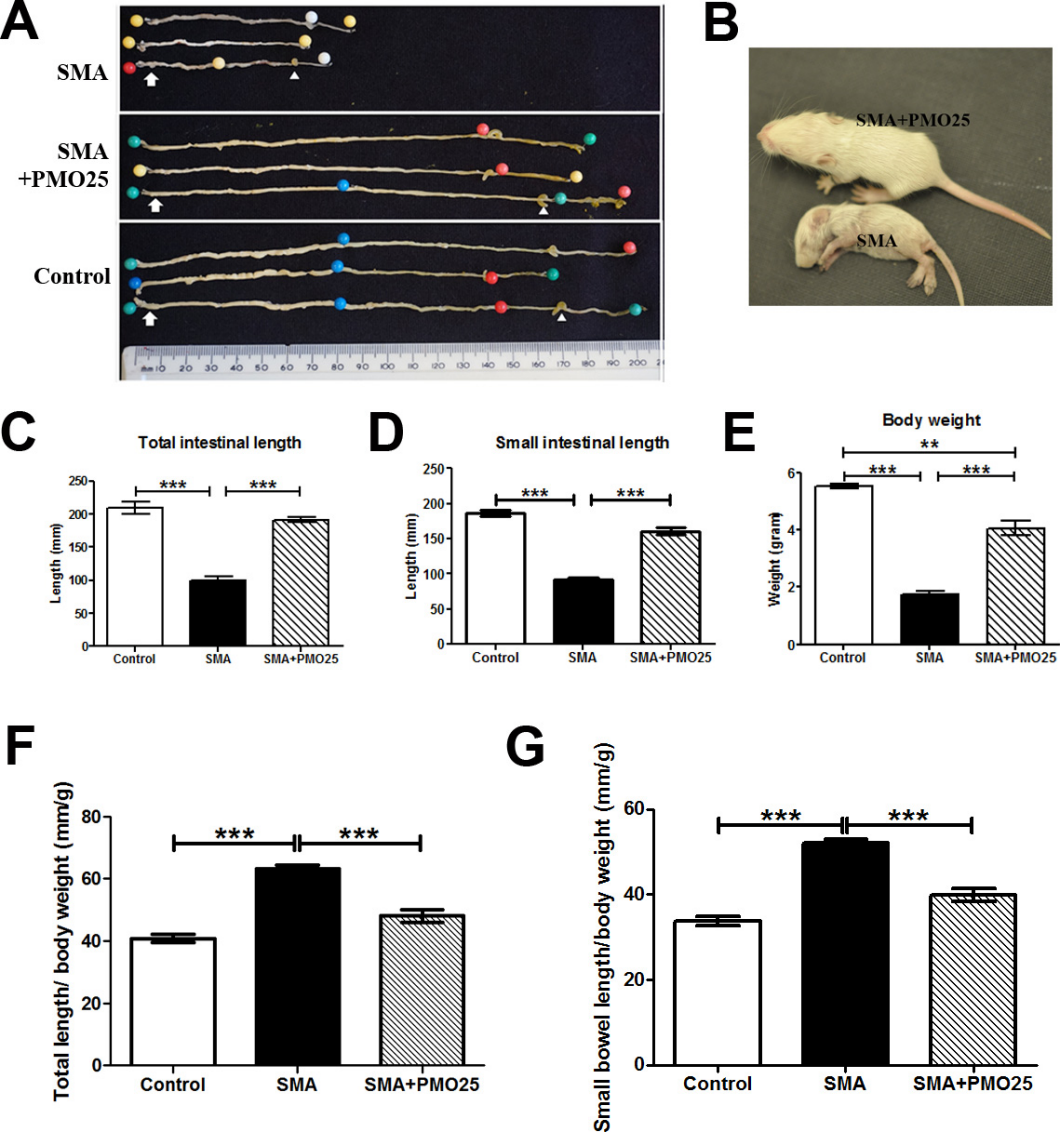
Gross anatomical features of the bowel in SMA, control and PMO25 treated SMA mice. (A) Images of the whole bowel from 10 day old SMA (N = 3), control (N = 3) and PMO25 treated SMA mice (N = 3). Specimens were pinned in order to display the whole bowel length. Arrows indicate proximal duodenum and arrowheads identify the cecum. (B) Representative image of SMA and PMO25 treated SMA mice at PND10. (C) SMA mice had the shortest whole bowel length compared to control and PMO25 treated mice. (P< 0.0001 vs control and vs SMA+PMO25. N = 6–7). (D) The mean small bowel length was significantly lower in SMA mice than in control and treated mice (P< 0.0001 vs control and vs SMA+PMO25 in small bowel. N = 3–4). (E) SMA mice displayed the lowest body weight compared to control and PMO25 treated mice. (P< 0.0001 vs control and P<0.0001 vs SMA+PMO25. N = 3–4). The relative total intestine length to body weight (F) and relative small intestinal length to body weight (**G**) in SMA mice were significantly higher than those in control and PMO25 treated mice (P<0.0001 N = 3–4). ***P < 0.001, *P<0.05.

The severe SMA mice have extremely shortened lifespan and distinctly small body size. These phenotypes can be successfully rescued by systemic morpholino antisense oligomer treatment [10, 30]. To determine the general condition of the GI system in the severe SMA mice and the response to systemic PMO25 treatment, we measured the length of intestine and the body weight (Fig 1A and 1B). There was a significant reduction in the length (mm) of the intestine in SMA mice (total length: 99.71 ± 5.63 N = 7; small intestine: 91.25 ± 3.14 N = 4) compared to control mice (total length: 208.7 ± 9.28 N = 6; small intestine: 186.3 ± 4.33 N = 3; P<0.0001) (Fig 1A, 1C and 1D). There was also a significant reduction in body weight (gram) in SMA mice (1.75 ± 0.08 N = 4) compared to control mice (5.52 ± 0.07 N = 3; P<0.0001) (Fig 1E). However, the relative length of intestine to body weight (mm/g) in SMA mice (relative total intestinal length: 63.37 ± 1.17 N = 4; relative small intestinal length: 52.19 ± 0.81 N = 4) was significantly higher than those in control mice (relative total intestinal length: 40.86 ± 1.22 N = 3, P<0.0001; relative small intestinal length: 33.74 ± 1.05 N = 3, P<0.0001) (Fig 1F and 1G). This suggests that SMA mice, though very small, had disproportionately long intestines compared to control. After PMO25 treatment, both the body weight (4.06 ± 0.25 N = 4; P<0.001 *vs* SMA) and bowel length (total length: 191.0 ± 4.03 N = 7; small intestine: 161.0 ± 5.75 N = 4; P<0.001 *vs* SMA) were significantly restored to near-normal levels (Fig 1C, 1D and 1E). PMO25 treated mice also showed a significant reduction in the relative length of the full (48.07 ± 2.04 N = 4; P = 0.0006 *vs* SMA) and small intestine (39.91 ± 1.63 N = 4; P = 0.0005 *vs* SMA) (Fig 1F and 1G). This indicates that AON treatment has restored body weight and relative length of the intestines to near-normal values.

### Histopathological defects in the small intestine of SMA mice

**Fig 2:**
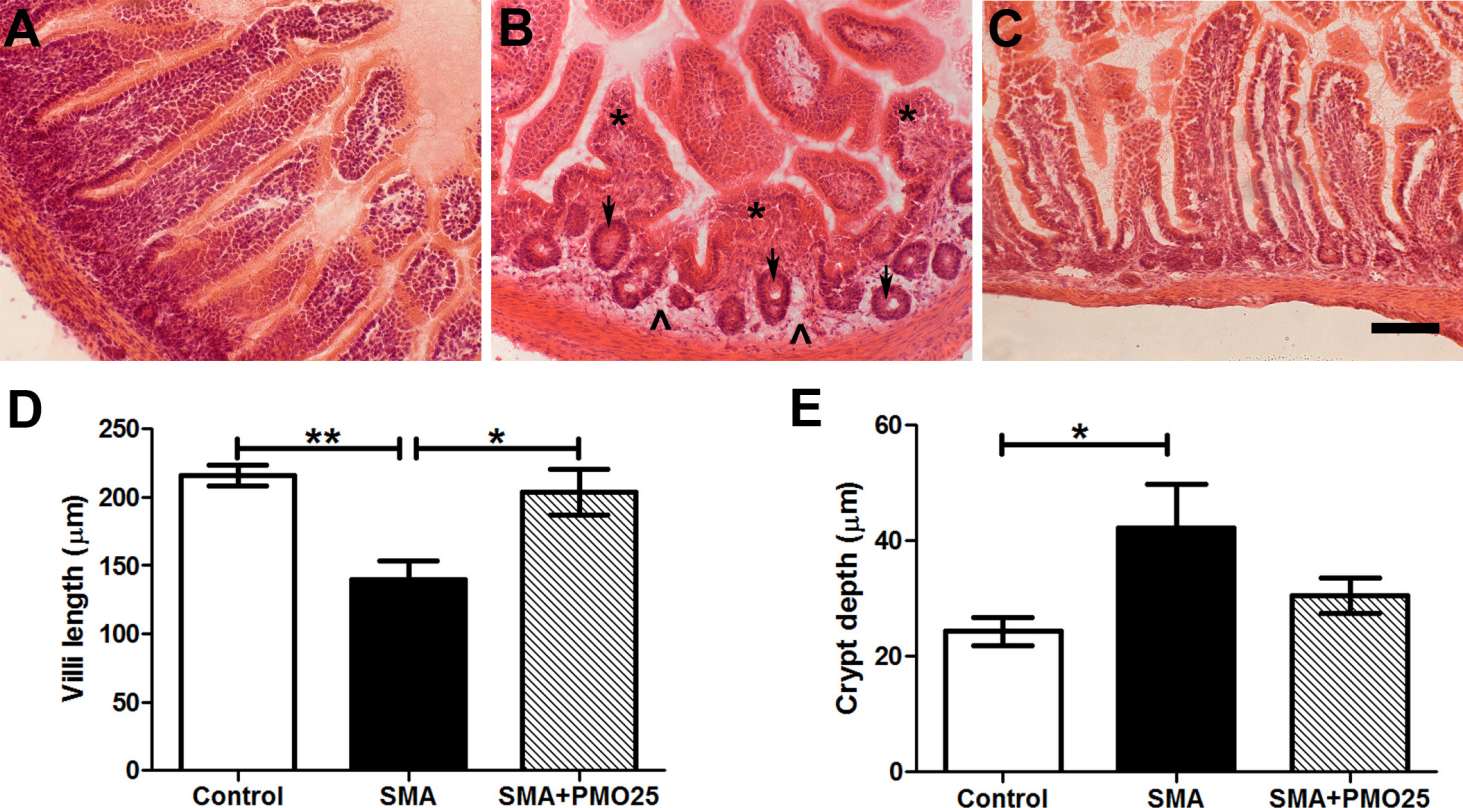
Histology of small intestine. H&E staining of (A) Control (B) SMA (C) SMA+PMO25 small intestine. Shortened and blunted villi (* asterisk) and intramural edema (^ arrow head) were present in the lamina propria layer in SMA mice, along with the distinct intestinal crypt architectural distortion (arrow). Quantification of the villus length (D) and crypt size (E) in mice. **P < 0.001, *P < 0.05. Scale bar = 100 μm.

We next carried out a histological evaluation of the microstructure of duodenum in H&E stained transverse sections. Distinct histopathological abnormalities were present in SMA mice compared to controls (Fig 2A and 2B), and were dramatically improved after systemic PMO25 treatment (Fig 2C). Villi, the characteristic finger-like projections of the wall of the small intestine, in SMA mice were blunted (Fig 2B) and significantly reduced in length (139.6 ± 13.95 μm, N = 3) compared to control mice (215.7 ± 7.59 μm, N = 3; P<0.01) (Fig 2D). Diffused edema of the lamina propria, which underlies the epithelial layer of the gut tube, was also noted in SMA intestine (Fig 2B). These observations are consistent with previous findings in the Taiwanese SMA mouse model [32]. In addition the intestinal crypts (which lie between and at the base of projecting villi) were irregular in size, shape and distribution in SMA mice (Fig 2B). The depth of crypt in SMA mice (42.19 ± 7.60 μm, N = 3) was significantly greater than in control mice (24.36 ± 2.43 μm, N = 3; P<0.05) (Fig 2E). After systemic PMO25 treatment, all the histopathological alterations described above were significantly improved, with the length of villi (203.6 ± 16.48 μm, N = 3; P<0.05 *vs* SMA; no significance *vs* control; Fig 2D) and depth of crypts (30.55 ± 3.00 μm, N = 3; no significance *vs* control; Fig 2E) significantly improved to near-normal levels.

### Significantly decreased vascular density in SMA mouse small intestine

**Fig 3:**
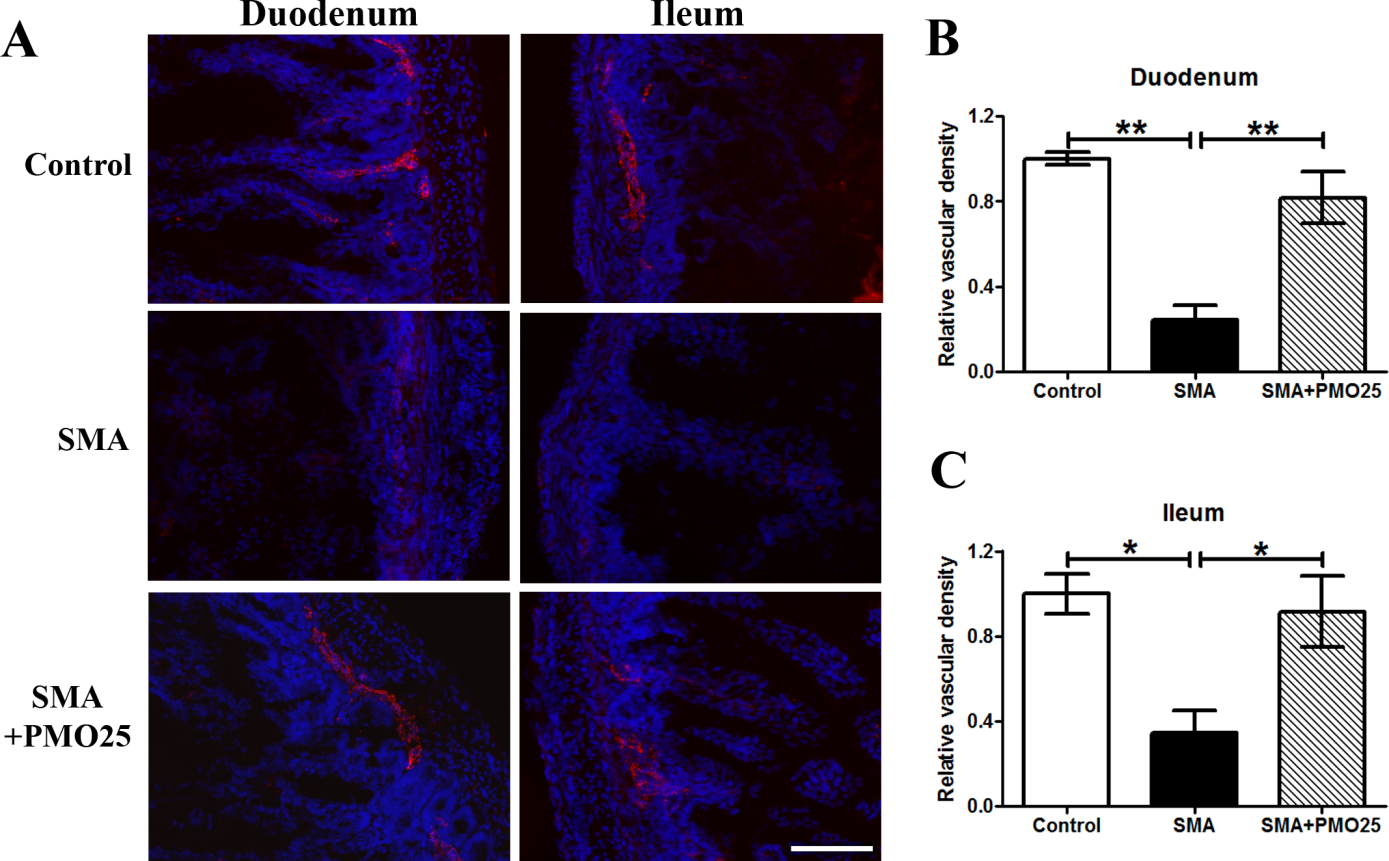
Blood vessel density in duodenum and ileum. (A) Representative image of vWF Immunofluorescence staining in duodenum and ileum of small intestine in control, SMA and PMO25 treated SMA mice. Blood vessels were indicated by vWF (red) staining. DAPI (blue) stains DNA nuclear and was used to outline the intestinal structure. Proportion of vascular density in duodenum (B) and ileum (C). The vascular density was quantified as pixels/unit area using imageJ software. Values in all three groups were then normalized to the mean value in the group of untreated SMA mice. Vascular density was significantly reduced in SMA mice in duodenum (P < 0.001 *vs* control, P <0.01 *vs* SMA+PMO25) and ileum (P < 0.05 *vs* control, P<0.05 *vs* SMA+PMO25), and was significantly improved after PMO25 treatment. (* P < 0.05, ** P<0.01). Scale bar = 50 μm.

We and others recently reported decreased vascular density in skeletal muscle and spinal cord in SMA mice [26,27] and in muscle from young SMA patients [27]. To assess the relationship between the involvement of vascular developmental abnormalities in the gut and its relationship to the observed gut phenotype in SMA, we measured the vascular density in the small intestine in SMA mice. Von Willebrand factor (vWF) immunofluorescence staining in duodenum and ileum (Fig 3A) revealed a dramatic decrease in vascular density in SMA mice. There was a significant reduction of blood vessel density in both duodenum (75% reduction) and ileum (65% reduction) in SMA mice compared to littermate controls (Fig 3B and 3C). The decreased vascular density in both duodenum and ileum was significantly improved in SMA mice after systemic PMO25 treatment (P<0.01 and P<0.05 *vs* SMA in duodenum and ileum, respectively), to near-normal levels (no significance between SMA+PMO25 and control; Fig 3B and 3C).

### Significant increase in enteric neuron numbers in SMA mouse small intestine

**Fig 4:**
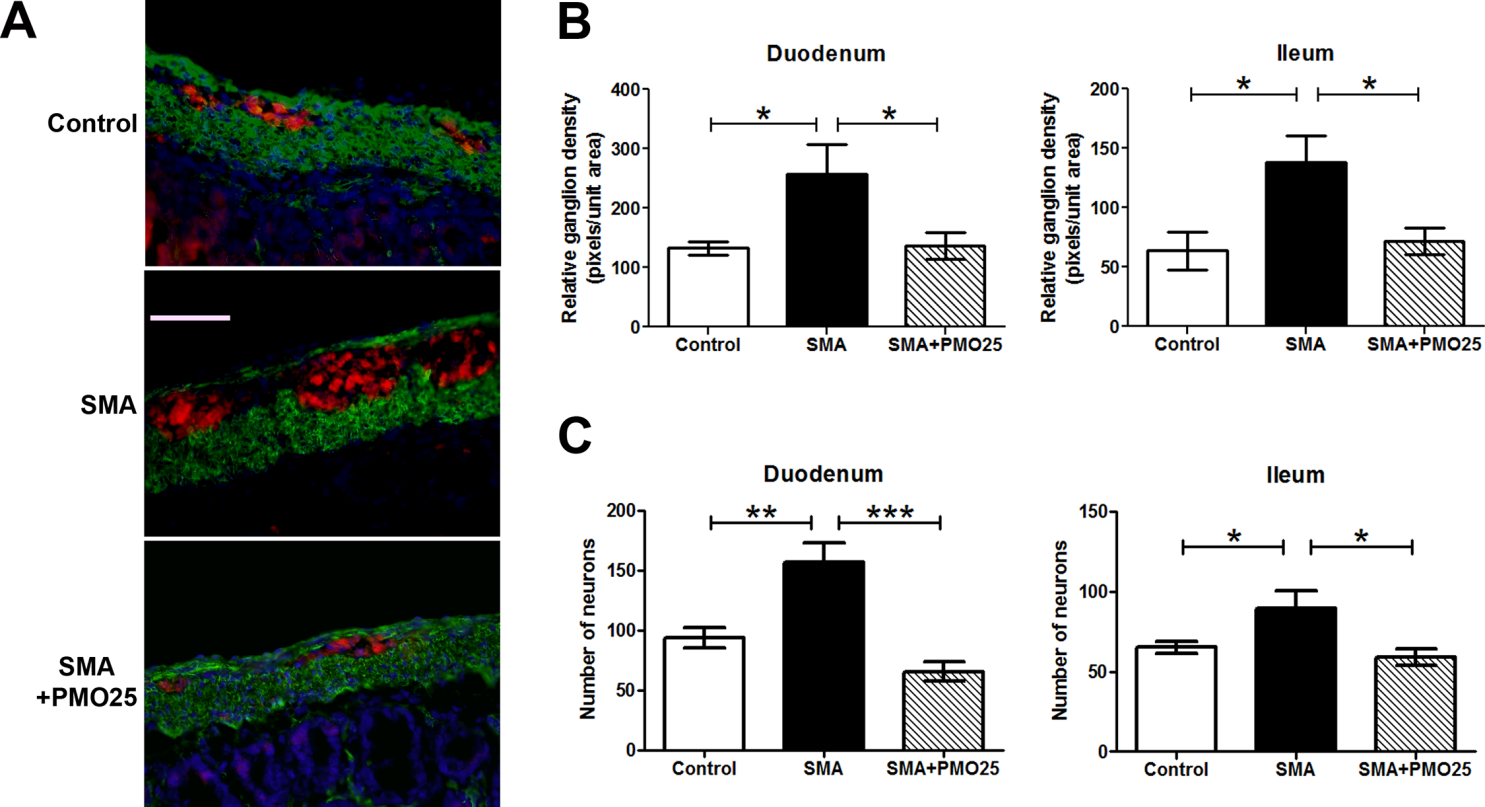
Enteric neurons in SMA mouse small intestine. (A) Representative image of enteric neurons/ganglions in Duodenum myenteric plexuses from control, SMA and PMO25 treated SMA mice. Enteric neurons were stained with neuronal marker PGP9.5 (red). The muscular layer was stained with α-smooth muscle actin (green). Cell nuclei were stained with DAPI (blue). (B) Relative ganglion density in 3 groups of mice. Pixels of PGP9.5 immunostaining per captured field was used to quantify the ganglion density using imageJ software and expressed as pixels per unit area. Ganglion density was significantly increased in SMA mice in both duodenum (P = 0.028 *vs* control, N = 4 per group) and ileum (P = 0.018 *vs* control, N = 4 per group) and significantly decreased after PMO25 treatment (P = 0.038 in duodenum; P = 0.019 in ileum; N = 4 per group). (C) The mean number of neurons was also significantly increased in both duodenum (P = 0.0045 *vs* control, P< 0.001 *vs* PMO25 treatment) and ileum (P = 0.04 *vs* control, P = 0.012 *vs* PMO25 treated SMA) in SMA mice and was reduced significantly by PMO25 treatment (N = 6–8, * P < 0.05; ** P < 0.01; *** P < 0.001). Scale bar = 25 μm.

The autonomic nervous system acting through the enteric nervous system (ENS) is responsible for the regulation and control of all gastrointestinal functions. Disrupted ENS signaling in intestine, resulting in defective GI function, has been reported in the adult stage of two mild mouse models of Smn deficiency [33]. In this study, we examined enteric neurons in the severe Taiwanese SMA mouse model, which has a severe phenotype and short lifespan. Ganglion density and neuron numbers in the myenteric plexuses were identified and quantified by PGP9.5 staining. To identify the muscular layer of the intestine wall, α-smooth muscle actin staining was used to discriminate myenteric from submucosal plexuses. Unexpectedly, the area occupied by ganglia of the myenteric plexus was significantly increased in SMA mice with more closely-packed neurons compared to control mice (Fig 4). The number of neurons and ganglion density in the myenteric plexuses were significantly greater in SMA compared to control mice, in both duodenum (ganglion density: 256.7±51.6 *vs* 132.7±10.7, N = 4, P<0.05; neurons: 156.9±16.3 *vs* 93.8±8.3, N = 6–8, P<0.01) and ileum (ganglion density: 137.9±22.6 *vs* 63.8±16.0, N = 4, P<0.05; neurons: 89.4±10.9 *vs* 65.1±3.6, N = 6–8, P<0.05). The ganglion density and number of neurons were significantly reduced to near-normal levels after PMO25 treatment in both duodenum (ganglion density: 256.7±51.6 (SMA) *vs* 136.5±21.9 (SMA+PMO25), N = 4, P<0.05; neuron number: 156.9± 16.3 *vs* 65.4± 8.1, N = 8, P<0.001) and ileum (ganglion density: 137.9±22.6 and 71.8±11.0, N = 4, P<0.05; neuron number: 89.4±10.9 vs 59.0±5.4, N = 8, P<0.05). There was no significant difference between all parameters in control and SMA+PMO25 mice (Fig 4).

### Increased macrophage infiltration in intestine of SMA mice

**Fig 5:**
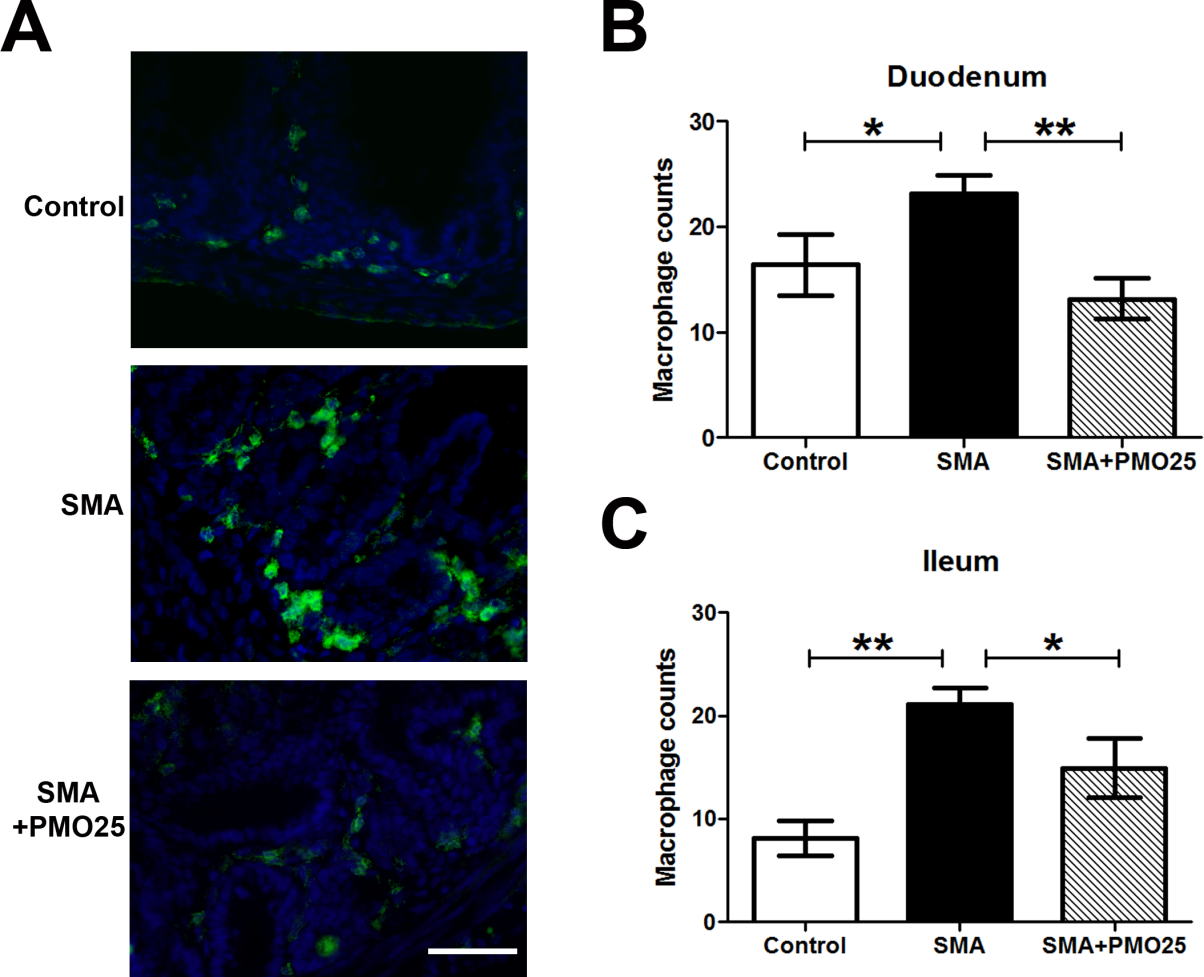
Increased macrophage infiltration in the gut of SMA mouse. (A) Representative image of macrophage staining in duodenum segment in SMA, control and PMO25 treated mice. Macrophages were stained with F4-80 antibody (green) and nuclei were stained with DAPI (blue). The absolute macrophage numbers per area in duodenum (B) and ileum (C) in three groups of mice. (N = 6. * P < 0.05; ** P < 0.01). Scale bar = 25 μm.

An excess of enteric neurons has been reported in patients with inflammatory bowel disease [34,35]. The correlation between enteric neuron density and the severity of intestinal inflammation has also been indicated in transgenic mice with aberrant numbers of neurons in the ENS [21]. To understand if the increased number of enteric neurons in the intestine of severe SMA mice is correlated with inflammation, macrophage infiltration was examined in duodenum and ileum segments from SMA, control and PMO25 treated SMA mice. Significantly increased macrophage numbers were observed in the intestine of SMA mice (23.20 ± 1.74, N = 6 in duodenum; 21.05 ± 1.69, N = 6 in ileum) compared to control mice (16.40 ± 2.91, N = 6 in duodenum, P<0.05; 8.10 ± 1.65, N = 6 in ileum, P<0.01; Fig 5). The numbers were significantly reduced to near-normal levels after PMO25 treatment (13.17 ± 1.93, N = 6 in duodenum, P<0.01 *vs* SMA; 14.92 ± 2.90, N = 5 in ileum, P<0.05 *vs* SMA). There was no significant difference in macrophage numbers between control and SMA+PMO25 mice.

### Systemic administration of PMO25 augments exon 7 inclusion and restores SMN protein in intestine of SMA mice

**Fig 6:**
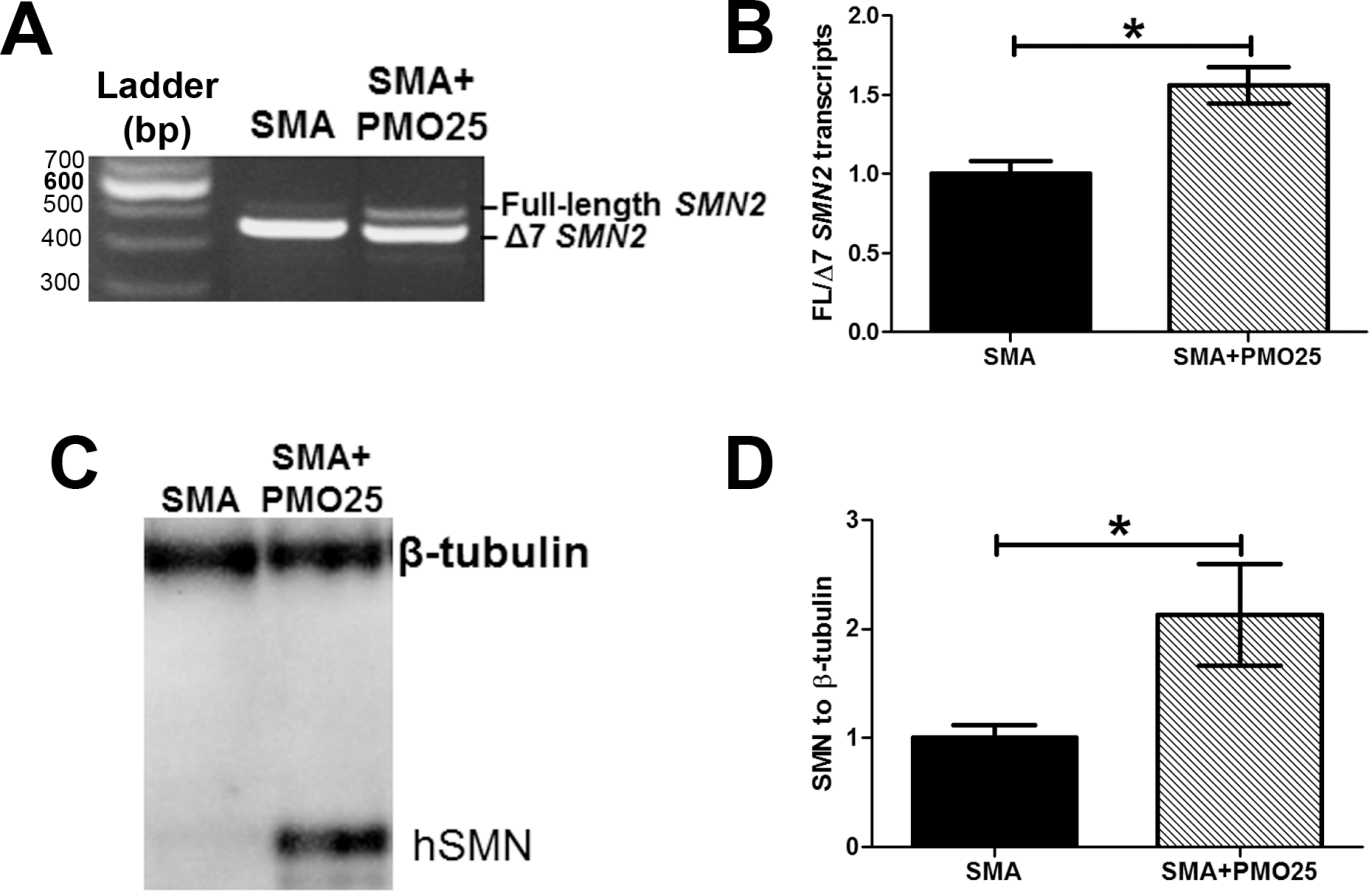
Systemic delivery of PMO25 increased *SMN2* exon 7 inclusion and SMN protein expression in intestine. (A) Representative image of reverse transcriptional polymerase chain reaction (PCR) showed the partial increase of full-length *SMN2* in SMA mice after PMO25 treatment. (B) Quantitative real-time PCR of full-length *SMN2* to Δ7 *SMN2* transcript ratio. (C) Western blotting assay of human SMN protein in intestine tissues from SMA and PMO25 treated SMA mice. β–tubulin was used as loading control. (D) Semi-quantification of SMN protein relative to tubulin control. Data were normalized to the ratio of SMN/tubulin in untreated SMA mice. (N = 3, *P< 0.05)

We have previously shown that systemic administration of PMO25 successfully rescues severe SMA mice [10]. A significant increase in *SMN2* exon 7 inclusion and SMN protein were detected in the central nervous system after PMO25 was delivered subcutaneously in severe SMA mice on PND0, in keeping with the incomplete blood brain barrier function in newborn mice [10]. In addition, we have also demonstrated that regular systemic administration of low-dose PMO25 at later stage can still benefit SMA mice with intermediate phenotypes, and suggest that restoration of SMN in peripheral systems, in addition to CNS restoration, is important for SMA treatment [30]. To determine the efficiency of AON therapy on SMN expression in intestine, *SMN2* exon 7 inclusion and the expression of SMN protein in duodenal segments were assessed by quantitative real-time PCR and western blotting, respectively. The ratio of full-length *SMN2* to Δ7 *SMN2* transcripts in intestine was significantly increased after systemic PMO25 treatment (P = 0.014; Fig 6A and 6B). SMN protein was increased approximately 2-fold in intestine after PMO25 treatment (Fig 6C and 6D).

## Discussion

Here we describe detailed morphological changes in a severe mouse model of SMA, including gross and microscopic structural changes in addition to an unexpected increase in the extent of the enteric nervous system and in inflammation identified by macrophage infiltration of the small intestine. In each case, these significant structural, and likely functional, defects in the small intestine are restored to near-normal values by systemic treatment with an AON designed to increase *SMN2* exon 7 inclusion.

The striking morphological changes in the small intestine of SMA mice—shortened and blunt villi, intramural edema in the lamina propria layer of the intestinal wall and enlarged intestinal crypts (Figs 1 and 2)—are consistent with a previous report of pathological features in the intestine of severe SMA mice [36]. We were surprised to find that the relative length of intestine to body weight in SMA mice was longer than expected. This may be due to the steep body weight loss during the end stage of the rapidly progressive disease in mice.

Due to limitations related to the severe phenotype and short lifespan, we did not perform the GI functional assays in the severe SMA mice. However, studies in less severe adult SMA mice have already clearly demonstrated the direct impact of Smn deficiency on GI function [33]. Despite normal activity levels and food and water intake, transgenic mice with nestin-cre-mediated recombination of mouse *Smn* on the SMNΔ7 background had constipation, delayed gastric emptying, slow intestinal transit and reduced colonic motility [33]. Malnutrition and GI dysmotility are also a common manifestation in SMA type I and II patients [20,37]. The intestinal problems in SMA patients have been proposed to result from GI tract smooth muscle weakness secondary to the inadequacy of the autonomic ENS [18]. However, evidence from our study suggests another possibility: that impaired GI function may be associated with a reduction in vascular density and an increase in inflammation in the intestine.

Decreased vascular density has been reported in skeletal muscle and spinal cord in severe mouse models of SMA [26,27]. In patients, vascular defects such as digital necrosis and distal vascular thrombosis have been reported in severe SMA infants [22,23]. There is reduced vascular density in skeletal muscle in severe SMA patients and the vascular system in muscle fails to develop with age [27]. In this study, we show a striking reduction in vascular density in the small intestine of severe SMA mice (Fig 3). These mice presented significant weight loss, malnutrition, reduced mobility and diarrhea at the end stage of their lives, resembling the clinical presentation of patients with rare chronic mesenteric ischemia [38]. This appearance could therefore be linked to tissue hypoxia due to vascular defects, which was suggested in our previous studies in spinal cord in severe SMA mice [27]. Close interactions between the enteric nervous and vascular systems are indicated *in vitro* in cell culture assay of vascular cells and ENS-derived cells, *in vivo* in tyrosine kinase receptor RET knockout enteric ganglia deficiency mice and in human Hirschsprung’s disease [39]. Interestingly, contradictory data exists from mouse models and patients with enteric ganglia deficiency: with a reduction of blood vessel density in the RET knockout mouse model, but more blood vessels detected in the enteric aganglionic zone of patients with Hirschsprung’s disease [39]. We report similar inconsistencies in vascular density and enteric neuron numbers between SMA mice and patients. While the numbers of enteric neurons and ganglion density in duodenum and ileum were dramatically increased in SMA mice (Fig 4), a study conducted in 8 infants with Werdnig-Hoffmann disease (SMA type I) shows low values of neural tissue in myenteric plexus in small intestine and colon [40].

The increased numbers of enteric neurons found in severe SMA mice could be either a compensatory mechanism to help maintain GI function, or a response to other underlying pathogenic changes. Overabundance of enteric neurons has been reported in inflammatory bowel disease [34,35], where macrophage infiltration is implicated in disease pathogenesis [41]. We found a significant increase in numbers of macrophages in small intestines of SMA mice (Fig 5). The severity of intestinal inflammation has been shown to be associated with the density of the enteric neurons in transgenic mice with either hyperplastic or hypoplastic ENS [21]. We show a similar correlation between enteric neuron overabundance and increased inflammation in intestine in SMA mice. It is therefore tempting to speculate that the overabundance of enteric neurons and ganglia may be a response to the increased macrophage infiltration in the intestine in SMA mice.

Importantly, we show that histopathological abnormalities in intestine in severe SMA mice are significantly improved by systemic PMO25 treatment. The morpholino antisense oligomer PMO25 has been previously reported by our group to effectively augment *SMN2* exon 7 inclusion and restore SMN protein in SMN deficiency mouse models [10,30]. Systemic administration of a single 40 μg/g dosage on PND0 completely rescues severe SMA mice by increasing the lifespan from 10 days to over 200 days. Significant restoration of SMN was detected in brain and spinal cord after the systemic delivery of PMO25 in newborn SMA mice when the blood brain barrier is still penetrable [10]. In addition, chronic systemic administration of PMO25 at a later adult stage in SMA mice with intermediate phenotypes also showed the beneficial effect on disease progression [30]. The latter study also indicates the involvement of peripheral organs in disease pathogenesis. In this study we extend the involvement of the GI system in SMA and demonstrate its responsiveness to systemic AON treatment.

By analyzing *SMN2* exon 7 inclusion and SMN protein expression in intestine in SMA mice, we have shown that systemic PMO25 administration directly targets *SMN2* exon 7 splicing in intestine. This provides new insight into the biodistribution of PMO25 *in vivo*. While SMA is widely accepted as a lower motor neuron disease with spinal motor neurons being the primary pathological target, the involvement of additional peripheral organs has been implicated to contribute to the pathogenesis of the disease [18,19,42]. It is becoming increasingly important to understand in which organs there is a requirement for SMN protein, as interventions acting exclusively on the CNS will not address the peripheral manifestations of the disease and could affect long term outcomes in some ongoing clinical studies, especially in severe type I SMA infants.

In conclusion, we have shown significant histopathological abnormalities in intestine of SMA mice and that these abnormalities are reversed to near-normal by systemic PMO25 treatment. We speculate that enteric vascular defects, secondary to SMN deficiency, are responsible for the pathological changes in the intestine. This study provides crucial insight into potential additional therapeutic targets and will facilitate the development of future therapies.
